# Investigation of *VSX1* sequence variants in South Indian patients with sporadic cases of keratoconus

**DOI:** 10.1186/1756-0500-6-103

**Published:** 2013-03-18

**Authors:** Anshuman Verma, Manoranjan Das, Muthiah Srinivasan, Namperumalsamy V Prajna, Periasamy Sundaresan

**Affiliations:** 1 Department of Genetics, Dr. G. Venkataswamy Eye Research Institute, Aravind Medical Research Foundation, Aravind Eye Hospital, Madurai, Tamil Nadu, India; 2 Cornea Clinic, Aravind Eye Hospital, Madurai, Tamil Nadu, India

**Keywords:** KTCN - Keratoconus, *VSX1 -* Visual system homeobox 1, SNP - Single nucleotide polymorphism

## Abstract

**Background:**

The involvement of *VSX1* gene for the genetic basis of keratoconus is unclear and controversial. The genetic screening of *VSX1* from different ethnic populations can enlighten this subject. The aim of the present study is to investigate the role of *VSX1* gene in patients with sporadic cases of keratoconus from South India.

**Methods:**

The *VSX1* gene coding regions, including exon-intron boundaries were screened by direct sequencing analysis in 117 sporadic cases of keratoconus. The identified variations were also analyzed in 108 ethnic matched healthy blood donors.

**Results:**

In the *VSX*1 gene screening, no pathogenic mutation was identified, whereas we could find the presence of four reported single nucleotide polymorphisms; c.546A>G (rs12480307), c.627+23G>A (rs6138482), c.627+84T>A (rs56157240) and c.504-24C>T (IVS3-24C)*.* These variations were observed in similar frequency between cases and controls.

**Conclusions:**

The lack of *VSX1* pathogenic variations in a large number of unrelated sporadic keratoconus patients tend to omit its role, and corroborate the involvement of other genetic, environmental or behavioural factors in the development of this complex disorder.

## Background

Keratoconus (KTCN; [OMIM] 148300) is a corneal ectatic disorder characterized by progressive thinning of the central cornea, which acquires a conical shape rather than its normal dome-shaped curve. This results in optical aberrations leading to distorted blurred vision, progressive high myopia and irregular astigmatism [1]. Keratoconus is mostly bilateral but can be unilateral, onset in teenage years and often progresses until the third or fourth decade of life [2]. The prevalence of keratoconus is estimated to be 1 in 2000 in the general population [2,3]. It is more common in patients with atopic conditions, such as asthma, dermatitis, and found to be associated with many other genetic disorders like Down syndrome, posterior polymorphous corneal dystrophy, Leber congenital amaurosis, retinitis pigmentosa, Marfan syndrome, mitral valve prolapse and other documented associations [1,2,4]. It occurs usually in a sporadic form, though positive familial history has been reported in 6-10% of patients, with mostly autosomal dominant and sometime recessive or X-linked mode of inheritance [4-6]. Common histopathological features of KTCN include stromal thinning, iron deposition in the epithelial basement membrane, breaks in corneal Bowman’s layer and sometimes endothelial damage [2,7]. The disease generally occurs with no gender preponderance; however, few evidence suggests a higher prevalence in either male or female [1,6,8]. At present, the milder forms of keratoconus are corrected by spectacles or contact lenses. In 10% to 20% of KTCN cases, corneal thinning may reach such a severity that it becomes very essential to undergo corneal transplantation or penetrating keratoplasty, which has emerged as a major medical burden in many countries [2,9]. The exact etiology of KTCN is still unknown, and its pathogenesis may involve genetic as well as environmental or behavioural factors, such as ultra-violet radiation UVB, atopy/allergy, contact lens wear and mechanical eye rubbing, etc. [10-12]. Primarily, the genetic factors may play an important role in the development of keratoconus as evidenced by studies of familial inheritance, concordance between monozygotic twins and association with other known genetic disorders [12-14]. By linkage analysis and association studies, various loci have been mapped in families from different ethnic populations, well summarized by Nowak DM et al. [12]. Based on associated loci, genes such as *SPARC, LOX, TIMP3, COL6A1, COL8A1, MMP9,* and *MMP2,* etc., have been examined for their involvement [12,15]. The *SOD1* and *CRB1* were also analyzed as possible candidate genes because of their role in keratoconus associated disease like Down syndrome and Leber congenital amaurosis respectively [16,17]. However, genes under such studies, have not provided enough evidence for being an appropriate candidate gene in the pathogenesis of KTCN. One of the well-studied genes in genetic association with keratoconus is *VSX1*. Human *VSX1* [OMIM 605020] is a member of the *VSX1* group of vertebrate paired-like homeodomain transcription factors localized to human chromosome 20p11-q11. Heon et al., [18] first identified *VSX1* mutations in patients with either keratoconus or posterior polymorphous corneal dystrophy (PPCD). This led to the assumption that mutations in the *VSX1* gene may be involved in pathogenesis of keratoconus. A number of other studies, further showed the presence of *VSX1* variants in keratoconus patients from different ethnic populations [19-24]. Among them, several variants were found in highly conserved residues of *VSX1,* and predicted to be pathogenic by the bioinformatics tools like PolyPhen, SIFT, etc. [21,23]. The exonic and intragenic polymorphisms were frequently reported in such studies, and found to be associated with the disease in a few cases [22,25]. On the other hand, many other studies excluded those previously reported *VSX1* variations to be pathogenic, and showed a lack of mutation or association [26-30]. Therefore, the role of *VSX1* in KTCN pathogenesis is ambiguous, and further genetic studies are required for any persuasive conclusions. In this study, we have undertaken the sequence analysis of coding regions of *VSX1* gene in order to determine its genetic involvement in South Indian patients with sporadic form of keratoconus.

## Methods

### Clinical assessment

Patients affected with keratoconus were recruited from cornea clinic, Aravind eye hospital, Madurai, between the period of 2009 to 2011. The study consisted of 117 unrelated sporadic keratoconus patients, including 69 males and 48 females with an age range between 12 to 46 years old and mean age 23.1 years (SD ±6.1 years). A total of 108 ethnic-matched healthy blood donors with age range between 17 to 65 years old and mean age 24.6 years (SD ±9.4), including 27 males and 81 females, with no major ocular disorders were taken as controls. Any KTCN subjects with co-existing allergy/atopy, or systemic diseases/syndromes such as Down syndrome, Leber congenital amaurosis, Ehlers-Danlos syndrome, osteogenesis imperfecta and pellucid marginal degeneration, or post-LASIK or other refractive surgeries were excluded from this study. All study subjects belonged to South Indian ethnicity, mainly from the region of Tamil Nadu. Informed consent was obtained from each of the subjects. The study obeyed the tenets of the declaration of Helsinki and was approved by the institutional review board of Aravind eye hospital, Madurai, Tamil Nadu, India. The diagnosis of KTCN was based on the presence of important clinical features such as Munson’s sign, Vogt’s striae, Fleischer ring, prominent corneal nerves, corneal scarring, etc. The orbscan II parameters, (anterior float, posterior float, keratometry and pachymetry reading) operated with software version 3.12 (Bausch & Lomb Inc., Rochester, NY) were taken for the final diagnosis using following measurements (a) ratio between the radius of anterior best-fit sphere (BFS) and posterior BFS >1.27 (b) power of posterior BFS >55D (c) difference between highest and lowest posterior float points >100 μ (d) corneal thickness index (CTI) >1.16 (e) irregularity at 3 mm zone >1.5D; 5 mm zone >2.5D and (f) K reading >47D. The patients diagnosed with forme-frusta keratoconus were not included in the study. Intra ocular pressure using pulsair non-contact tonometry was also taken for all patients. The study involved only sporadic keratoconus cases, and patients with a family history of KTCN were excluded. Furthermore, to confirm the sporadic nature of cases, three to four immediate available family members of each patient were clinically examined.

### DNA extraction and PCR amplification

Genomic DNA was isolated from peripheral blood leukocytes using the salt precipitation method as described by Miller et al., [31]. The primer pairs used to amplify each of the five coding *VSX1* exons were previously described [19]. PCR reaction (Eppendorf mastercycler, Westbury, NY) was carried out in a 20 μl reaction mixture set up containing 2 μl of 10X PCR buffer with 1.5 mM MgCl_2_, 200 mM dNTP, 0.2 mM of each forward and reverse primer, 0.2 U Taq DNA polymerase (Sigma) and 50 ng genomic DNA. The genomic DNA underwent initial denaturation for 5 min at 95°C, followed by 35 cycles at 94°C for 1 min, respective exons annealing at 58°C (ex1), 59°C (ex2), 62°C (ex3,4,5) for 1 min, extension at 72°C for 30 sec and final extension for 5 min at 72°C. The *VSX1* gene coding regions with their exon-intron junctions were examined by bidirectional sequencing analysis.

### DNA sequencing

The PCR products were pooled and purified using the gel-elution kit method (Bio Basic Inc. Canada). The purified PCR products were sequenced bidirectionally using Big Dye Terminator ready reaction mix and analyzed on an ABI-3130 genetic analyzer (Applied Biosystems, Fostercity, CA). The sequence data analysis was done using BLAST software and compared with the published nucleotide sequence of the *VSX1* gene [Gen Bank accession number NM_014588]. The identified variations were evaluated using Alamut software version 2.1e (Interactive Biosoftware, Rouen, France). The nomenclature, location and classification of variations were done based on the Alamut output.

### Comparative analysis

A comparative statistical analysis of genotype and allele frequency was done using chi-square or Fisher’s exact test in order to assess differences in the distribution of *VSX1* polymorphism between cases and controls. The allelic p-value and odds ratio was determined for each of the identified *VSX1* variants.

## Results & discussion

A total of 117 sporadic KTCN patients were analyzed for coding and flanking intronic regions of *VSX1* through bidirectional DNA sequencing analysis. In the *VSX1* gene screening, no pathogenic mutations were identified whereas, four reported single nucleotide polymorphisms *c.546A>G* (rs12480307), c.627+23G>A (rs6138482), c.627+84T>A (rs56157240) and c.504-24C>T (IVS3-24C) could be observed (Table 1). Further, these four SNPs were investigated in 108 ethnic-matched healthy controls, and their genotype and allele frequency was compared between cases and control. The comparative statistical analysis of allele frequency of these SNPs indicated similar distribution in both patient and control groups (Table 2). The polymorphism *c.546A>G* (rs12480307) was found in exon 3 encoding a synonymous alanine substitution at 182 amino acid position, which is highly conserved throughout many species. This variation was seen in 43 cases (36 heterozygous and 7 homozygous) and 32 controls (28 heterozygous and 4 homozygous). The statistical analysis showed no significant difference of allelic distribution (p value 0.205) among cases and controls. The polymorphic variant c.627+23G>A (rs6138482) was found in 29 cases (26 heterozygous and 3 homozygous) and 41 controls (40 heterozygous and 1 homozygous). The statistical analysis showed the allelic p value 0.099, which was insignificant. The polymorphic variant c.627+84T>A (rs56157240) was found in 58 cases (49 heterozygous and 9 homozygous) and 50 controls (43 heterozygous and 7 homozygous) with no significant difference of allelic distribution between case and control groups (p value 0.595). Another variant IVS3-24C>T (c.504-24C>T) was identified in 7 cases (7 heterozygous and 0 homozygous) and 7 controls (7 heterozygous and 0 homozygous) signifying its equal distribution (allelic p-value 0.879) between cases and controls. This change was recently reported by Mukesh Tanwar et al., [25] as a novel *VSX1* variant, and registered in GenBank [Accession number : GU471016].

**Table 1:** ***VSX1 *sequence variants observed in the study**

**SNP ID**	**VSX1 transcript ID**	**c.DNA Change**	**VSX1 Protein ID**	**Amino acid change**
rs12480307	NM_014588	c.546A>G	NP_055403	p.A182A
rs6138482	NM_014588	c.627+23G>A	NP_055403	…
rs56157240	NM_014588	c.627+84T>A	NP_055403	…
(IVS3-24C)	NM_014588	c.504-24C>T	NP_055403	…

**Table 2:** **Frequencies of *VSX*1 gene variants in sporadic KTCN cases and healthy controls**

**VSX1 gene variations**			**Case**		**Control**		**Allelic p value**	**OR (95% CI)**
			**n=117**	**%**	**n=108**	**%**		
(rs12480307) c.546A>G	Genotype	AA	74	63.2	76	70.4		
		GA	36	30.8	28	25.9		
		GG	7	6.0	4	3.7		
	Allele	A	184	78.6	180	83.4	0.205	0.74 (0.44-1.21)
		G	50	21.4	36	16.6		
(rs6138482) c.627+23G>A	Genotype	GG	88	75.2	67	62.0		
		GA	26	22.2	40	37.0		
		AA	3	2.6	1	0.9		
	Allele	G	202	86.3	174	80.6	0.099	0.66 (0.38-1.12)
		A	32	13.7	42	19.4		
(rs56157240) c.627+84T>A	Genotype	TT	59	50.4	58	53.7		
		TA	49	41.9	43	39.8		
		AA	9	7.7	7	6.5		
	Allele	T	167	71.4	159	73.6	0.595	1.12 (0.72-1.73)
		A	67	28.6	57	26.4		
IVS3-24C>T c.504-24C>T	Genotype	CC	110	94.0	101	93.5		
		CT	7	6..0	7	6.5		
		TT	0	0	0	0		
	Allele	C	227	97	209	96.7	0.879	1.09 (0.32-3.69)
		T	7	3	7	3.3		

OR-odds ratio, CI- confidence interval, p-value less than 0.05 was considered as significant.

## Conclusions

In our study, we have assessed the role of *VSX1* by sequence analysis of its five exons in 117 sporadic KTCN patients. Our screening showed the absence of pathogenic variations whereas, four previously reported SNPs were observed. Since no pathogenic changes were detected, we compared the genotype and allele frequency of each of the identified polymorphisms between the disease cohort and healthy controls to access their possible disease involvement. However, allele frequencies of these identified SNPs were found in similar frequency between cases and controls confirming their non-pathogenicity. Other *VSX1* gene variations such as *p.D144E, p.L17P, p.N151S p.G160D, p.P247R, p.L159M, p.G160V, p.Q175H, p.R166W* and *p.H244R,* reported in different previous *VSX1* studies were not identified in our analysis [18-22,24,29]. There are few earlier studies, which have indicated the association of non pathogenic *VSX1* variations. Stabuc-Silih et al., [25] found an absence of *VSX1* pathogenic mutations but observed an association of c.650G>A polymorphism (p=0.043) in unrelated Slovenian patients diagnosed with the hereditary form of KTCN. Mok et al., [20] found a significant association of one *VSX1* intragenic polymorphism ‘IVS1-11’ in unrelated Korean keratoconus patients (p=0.001). Mutational screening of 66 unrelated patients with keratoconus (27 familial cases; 39 sporadic cases) from the European population, showed a minor role of *VSX1* in the pathogenesis of keratoconus [22]. However, several other studies have shown the absence of pathogenic variations or lack of association of *VSX1* variants with KTCN. Tang YG et al., [29] ruled out the association of previously reported *VSX1* variations in a case–control study of 77 white KTCN patients. Liskova et al., [28], in a study of 85 familial keratoconus pedigrees from different ethnic origins, found a lack of pathogenic variations in *VSX1* and disqualified the previously reported c.432C>G *(p.D144E)* change to be pathogenic. Aldave et al., [27] found the absence of mutation in 100 unrelated KTCN subjects and concluded that *VSX1* mutations are not associated with keratoconus. Overall, our study also rules out the possible involvement of *VSX1* gene in sporadic, South Indian KTCN patients. However, few previous evidence of *VSX1* pathogenic variations and their association with disease, suggest that it is more likely to be involved in a smaller subset of the KTCN population. The role of *VSX1* variations in a minority of keratoconus patients may be influenced by its possible variable penetrance or pleiotropic effect in corneal tissue.

Our study supports the previous evidence of lack of pathogenic variations in *VSX1*, and corroborates the involvement of new genes, loci or any other genetic or environmental factors. Linkage analysis and association study are the two main approaches used to identify novel genes. Genome wide association study (GWAS) is a more useful approach as it is wide-ranging, unbiased and can be applied even in the absence of convincing indication regarding the function or location of the causal genes. The other genetic factors are needed to be investigated for KTCN pathogenesis. Abu-Amero et al., [32], analyzed *VSX1* chromosomal copy number variations (deletions/duplications) in a group of sporadic patients, who were excluded for *VSX1* mutations, and verified that such possible genetic changes are also not involved in keratoconus. Recent studies find that keratoconus corneas have signs of oxidative stress and high level of mitochondrial DNA damage [33]. More recent findings suggest that micro-RNA can be involved in the pathogenesis of keratoconus [34]. Therefore, mitochondrial genes and micro-RNA are the prospective emerging areas to explore in the context of other genetic factors related to keratoconus. Proteomic profiles in the KTCN corneas and tear have shown differential expression of several proteins, which may have possible role in the etiology of keratoconus [35-37]. Hence, genetic and proteomic approaches together can provide more useful information regarding disease etiology. For the genetic basis of keratoconus, other genetic factors, new chromosomal loci and genes are the subject of investigation to accomplish the better understanding of the pathogenesis of disease.

## Abbreviations

KTCN: Keratoconus; VSX1: Visual system homeobox 1; PCR: Polymerase chain reaction; SNP: Single nucleotide polymorphism; DNA: Deoxyribonucleic acid; LASIK: Laser-assisted in situ keratomileusis.

## Competing interests

ALCON has provided the financial support for this project. AMRF has provided the partial support for article processing charge.

## Authors' contributions

AV and PS carried out the molecular genetic studies, participated in the sequence analysis and drafted the manuscript. MD, MS and NVP equally participated in the recruitment and clinical diagnosis of patients, helped in the design and coordination of the study. All authors read and approved the final manuscript.
